# Muscle Strength and Cardiovascular Health in MASLD: A Prospective Study

**DOI:** 10.3390/medicina61020247

**Published:** 2025-02-01

**Authors:** Birgül Fatma Kumbaroğlu, Yasemin Hatice Balaban, Tülin Düger

**Affiliations:** 1 Faculty of Physical Therapy and Rehabilitation, Hacettepe University, Ankara 06100, Turkey; tulin.duger@hacettepe.edu.tr; 2 Faculty of Medicine, Department of Gastroenterology, Hacettepe University, Ankara 06100, Turkey; ybalaban@hacettepe.edu.tr

**Keywords:** MASLD, nonalcoholic fatty liver disease, muscle strength, sarcopenia, cardiorespiratory fitness, fatigue, depression

## Abstract

*Background and Objectives*: The pathogenesis of metabolic dysfunction-associated steatotic liver disease (MASLD) remains incompletely understood. However, recent studies highlight the interactions between muscle, liver, and adipose tissue. This study aimed to explore the relationships between clinical indicators of MASLD and sarcopenia, cardiorespiratory fitness, fatigue, and mood. *Materials and Methods*: The study involved 60 participants, including 28 healthy controls and 32 with MASLD, categorized into two disease subgroups: 15 with MASL and 17 with metabolic dysfunction-associated steatohepatitis (MASH). Participants completed an incremental speed shuttle walk test to evaluate cardiorespiratory fitness, a hand-held dynamometer assessment for appendicular muscle strength, and the timed up and go test for physical performance. Physical activity level, fatigue, quality of life, and emotional state were assessed using questionnaires. The test results were compared between groups and with disease characteristics. *Results*: MASL and MASH groups showed reduced cardiorespiratory fitness (*p* < 0.001). The knee extensors were significantly weaker in both MASL and MASH groups (*p* < 0.001 and *p* = 0.001, respectively). The MASH group reported higher levels of depression and negative health perception (*p* = 0.006 and *p* = 0.03, respectively). Muscle strength in patients with MASLD showed a significant negative association with depression (OR = −0.384, 95% CI: −3.10 to −0.74, *p* = 0.003), intrahepatic triglyceride content (OR = −0.287, 95% CI: −1.31 to −0.11, *p* = 0.023), and LDL (OR = −0.286, 95% CI: −0.02 to −0.33, *p* = 0.03). In contrast, a positive association was observed between VO_2_ and muscle strength (OR = 0.531, 95% CI 1.27 to 3.47, *p* < 0.001). *Conclusions*: This study suggests that muscle strength is linked to key metabolic parameters, such as hepatic fat, LDL levels, and aerobic capacity, that may contribute to the development and progression of MASLD. Interventions aimed at preserving or enhancing muscle strength in MASLD patients may be essential for preventing liver damage and improving metabolic health.

## 1. Introduction

Metabolic dysfunction-associated steatotic liver disease (MASLD), the most common cause of chronic liver diseases [1], encompasses a spectrum of conditions characterized by excessive fat accumulation in hepatocytes and is accompanied by at least one metabolic factor, such as obesity, hyperglycemia, hypertension, or dyslipidemia [2]. In recent years, the need for a change in disease terminology became apparent, and the term MASLD, along with updated diagnostic criteria, has gained general acceptance, replacing the previous term, non-alcoholic fatty liver disease (NAFLD). This spectrum includes two well-described phenotypes and several intermediate stages. Specifically, metabolic dysfunction-associated steatotic liver (MASL) is defined by simple fat accumulation and is typically considered benign, whereas metabolic dysfunction-associated steatohepatitis (MASH) represents a more severe form characterized by liver inflammation and advanced fibrosis, which can progress to serious liver disease [3]. MASH is characterized by excessive fat accumulation in hepatocytes, similar to MASL; however, the lipotoxicity observed in MASH triggers inflammatory pathways that result in oxidative stress, mitochondrial dysfunction, and hepatocyte apoptosis [4]. Unlike MASL, MASH leads to hepatocyte necrosis and fibrosis, accelerating liver damage and increasing the risk of cardiovascular diseases, systemic inflammation, and progression to cirrhosis or hepatocellular carcinoma [5]. The global prevalence of MASLD has risen by approximately 50% with the rise in cases of metabolic syndrome, obesity, and type 2 diabetes (T2DM) [6], now affecting more than one-third of adults [1]. MASLD is strongly linked (50–80%) to insulin resistance, type 2 diabetes, dyslipidemia, hypertriglyceridemia, and hypertension with over 90% of obese individuals with type 2 diabetes affected by MASH [7,8]. These factors, in conjunction with cardiovascular risk factors, facilitate the progression of liver pathology [8,9].

Skeletal muscles regulate adipokine profiles, suppresses inflammation, and maintains metabolic balance, crucial for counteracting pro-inflammatory cytokines like TNF-α and IL-1 released during metabolic dysfunction [10,11]. Impaired muscle function, as seen in sarcopenia, exacerbates systemic inflammation and metabolic instability, increasing the risk of insulin resistance, metabolic syndrome, type 2 diabetes, and atherosclerosis [12]. Sarcopenia destabilizes metabolic homeostasis by impairing energy metabolism and mitochondrial function, creating a vicious cycle of inflammation and metabolic imbalance [13]. MASLD contributes to sarcopenia through insulin resistance, systemic inflammation, and mitochondrial dysfunction [14], while lipotoxic stress accelerates muscle protein breakdown [15], further reducing muscle strength and mass [16]. In turn, sarcopenia worsens MASLD by increasing liver fat accumulation and reducing physical activity [17], with elevated myostatin levels promoting insulin resistance and mitochondrial dysfunction [18]. This bidirectional relationship intensifies both MASLD and sarcopenia progression, further affecting liver and muscle health [19,20]. Although sarcopenia is estimated to affect up to 35% of individuals with MASLD and contributes to disease progression [21,22], research on sarcopenia risk remains limited, highlighting the necessity of exploring its relationship with MASLD severity.

Sarcopenia involves the loss of muscle mass, strength, and function, which is typically associated with aging but is also observed in chronic diseases [23,24]. Muscle strength is the strongest predictor of adverse outcomes as it directly affects daily activities and often signals early functional decline [25]. Although handgrip strength is commonly used to assess muscle strength, it mainly reflects the strength of the upper extremities; thus, evaluating lower extremity strength is recommended for a more precise assessment of sarcopenia [26]. Previous studies have attempted to link sarcopenia in MASLD with markers such as urinary creatinine excretion [27], scales [19], or hand grip strength [28]. However, these methods have not yet gained widespread acceptance, and further investigation of new approaches is recommended to reduce diagnostic variability [22]. This study aims to assess sarcopenia in patients with MASLD through muscle strength measurements, establish new reference values for sarcopenia, and compare the severity of MASLD with CRF, fatigue, and emotional status.

## 2. Materials and Methods

### 2.1. Design and Study Population

This research is prospective, cross-sectional, and observational study approved by the Hacettepe University Health Sciences Research Ethics Committee under registration number SBA 23/017. The inclusion criteria were the following: participants aged between 18 and 65 years old, with healthy controls and having no history of liver diseases, and participants with MASLD having a diagnosis confirmed by magnetic resonance imaging-proton density fat fraction (MRI-PDFF) and MR elastography (MRE). The patients included in the study were followed up according to the diagnostic criteria for NAFLD set by the American Gastroenterological Association/Asia–Pacific Study Group, but since the spectrum of MASLD has been shown to overlap almost completely with NAFLD [3,29], and the patients included in the study met the diagnostic criteria for MASLD (see Supplementary Materials), it was decided to use the current terminology.

Individuals with any neurological, orthopedic, or mental disorders, chronic conditions affecting physical performance, or those using walking aids were excluded from the study. Additionally, obese individuals (BMI > 30) and those with T2DM were not included in the research due to confounding effects on the outcome measurements [12]. Participants who met the criteria were invited to the study, and all of them signed an informed consent form. During the study, a total of 72 individuals were assessed for eligibility, and 7 people did not meet the inclusion criteria; 5 people did not volunteer to participate in the study.

### 2.2. Demographic and Laboratory Data

Participants consisted of MASLD patients who visited the gastroenterology clinic between 2023 and 2024 for control and liver-healthy individuals who came to the hospital for other reasons or accompanied patients. Age, sex, BMI, comorbidities, and smoking status were recorded. The intrahepatic triglyceride content and liver stiffness values of the patients were obtained from MRI-PDFF and MRE results conducted within the last year, and disease duration was recorded from medical records. Current fasting plasma glucose, insulin, glycosylated hemoglobin (HbA1c), alanine aminotransferase (ALT), aspartate aminotransferase (AST), gamma-glutamyl transferase (GGT), high-density lipoprotein (HDL) cholesterol, low-density lipoprotein (LDL) cholesterol, triglycerides, and C-reactive protein (CRP) levels were also recorded.

CRF was measured using a maximal exercise test. To assess sarcopenia, appendicular muscle strength and physical performance tests were conducted. Additionally, questionnaires were used to evaluate physical activity levels, fatigue, quality of life, and emotional state. The primary outcome measurement was knee extension strength and peak VO_2_, a reliable indicator of CRF. Secondary outcome measurements included functional performance tests, physical activity levels, fatigue, quality of life, and emotional state.

### 2.3. Cardiorespiratory Fitness

The incremental shuttle walk test (ISWT) was used to assess the aerobic exercise capacity of participants. This test is progressive and maximal, with the speed increasing by 0.17 m/s every minute over a duration of 12 min. A 10 m course was established using two cones. All tests were conducted by one experienced physiotherapist (BFK). The test was terminated if participants could not maintain the required speed due to dyspnea or fatigue, or if they could not complete the shuttle within the allowed time for a second attempt. Heart rate, arterial blood pressure, perceived dyspnea, and leg fatigue were recorded before and after the test. The predicted maximum heart rate was calculated using the formula 220-age. It has been shown that the peak VO_2_, obtained from the ISWT, correlates strongly with the results of cardiopulmonary exercise testing (CPET) conducted on treadmills or cycle ergometers, both in individuals with chronic respiratory diseases and in healthy individuals. [30,31]. Participants’ peak VO_2_ thresholds were determined using the ISWT. Finally, the estimated peak VO_2_ values, determined by Bruce et al. [32] for sedentary individuals, were calculated using the following formulas: peak VO_2_ (men) = 57.8 − (0.445 × age); peak VO_2_ (women) = 42.3 − (0.356 × age).

### 2.4. Muscle Strength

Muscle strength was measured using a digital hand-held dynamometer (J-TECH, Medical Commander PowerTrack II, Salt Lake City, UT, USA). Tests were conducted by the same physiotherapist on appendicular muscle groups, including shoulder flexors, shoulder extensors, elbow flexors, elbow extensors, hip flexors, hip extensors, knee flexors, and knee extensors. Measurements adhered to the guidelines provided by Bohannon and the manufacturer’s instructions [33]. The maximum force generated during isometric contraction was recorded for each muscle. Measurements were conducted bilaterally for each muscle group, with three trials per group, and the arithmetic mean of these trials was recorded.

### 2.5. Physical Performance

Participants’ functional performance was assessed using the timed up and go (TUG) test, which is also employed to examine the impact of sarcopenia on functionality. During the test, participants were instructed to stand up from an armless chair, walk three meters, turn around, walk back, and sit down again. The time taken to complete the test was recorded in seconds using a stopwatch. The test was repeated three times, and the arithmetic mean of the results was calculated [34].

### 2.6. Physical Activity Level

The short form of the international physical activity questionnaire (IPAQ) was used to assess participants’ physical activity levels in daily life. As a self-reported tool, IPAQ divides activity levels into three categories: vigorous, moderate, and walking activities. It allows for the calculation of energy expenditure by multiplying the time spent on these activities by their metabolic equivalents. Additionally, it records the amount of time participants spend sitting during the day, expressed in minutes [35].

### 2.7. Fatigue

Participants’ fatigue was assessed using the fatigue severity scale (FSS). The scale consists of 9 items that evaluate fatigue levels over the past week. Each item is rated on a scale from 1 to 9, with higher scores indicating greater levels of chronic fatigue [36].

### 2.8. Quality of Life

The short form-36 (SF-36) questionnaire, a generic scale, was used to assess quality of life. It includes eight subdomains of quality of life: general health perception, physical function, social function, pain, mental health, role limitations due to physical health, role limitations due to emotional problems, and vitality. Each subdomain is scored from 0 to 100, with higher scores indicating better health status [37].

### 2.9. Emotional Status

Anxiety and depression were assessed using the hospital anxiety and depression scale (HADS). This fourteen-item scale combines a seven-item anxiety subscale (HADS-A) and a seven-item depression subscale (HADS-D). Each item is scored from 0 to 3 on a four-point Likert scale, resulting in scores ranging from 0 to 21 for each subscale [38].

### 2.10. Statistical Analysis

All data were analyzed using the Statistical Package for the Social Sciences (SPSS), Version 23.0 (SPSS Inc., Chicago, IL, USA). Data normality was assessed with the Shapiro–Wilk test. Variables with a normal distribution were presented as mean ± standard deviation (SD), non-normally distributed variables as median and interquartile ranges (IQR), and categorical variables as numbers (percentage). To better assess the relationship between disease severity and appendicular muscle strength and CRF, MASLD patients were divided into two groups: MASL and MASH. For between-group comparisons of categorical variables, the chi-square test or Fisher’s exact test was used. Comparisons between two groups were conducted using Student’s *t*-test or its nonparametric equivalent, and for three-group comparisons, one-way ANOVA or its nonparametric equivalent was used. Post hoc tests were employed for further analysis if differences between groups were found. To identify independent risk factors for sarcopenia, a multiple linear regression analysis was conducted with muscle strength as the dependent variable. Model validity was assessed through the correlation coefficient (R^2^), variance inflation factor (VIF), and Durbin–Watson and Cook’s analyses. There were no missing data. The significance level for all statistical analyses was set at *p* < 0.05. The sample size was calculated using G*Power^®^ version 3.1 based on a similar study examining absolute changes in peak VO_2_ between MASLD and healthy controls [39]. To achieve 95% power at a 5% significance level, a minimum sample size of 40 participants in total was required. With subgroup analysis based on MASLD severity, the target sample size was set at 60 participants.

## 3. Results

### Demographic Characteristics

Sixty participants were included in the control (n = 28) and MASLD (n = 32) groups. Patients with MASLD were further subdivided into two groups: MASL and MASH, while healthy controls comprised the third group. There were no statistically significant differences among the groups in terms of age, gender, smoking status, or comorbidities (Table 1). Regarding insulin resistance, it was observed in 3 participants (11%) in the control group, 7 participants (46%) in the MASL group, and 4 participants (23%) in the MASH group. In addition, the BMI of patients with MASLD and MASH was significantly higher than that of the healthy controls (*p* < 0.001).

The biochemical and radiological findings for patients with MASLD were consistent with their diagnoses (Table 2). According to MRI-PDFF results, liver stiffness was significantly higher in the MASH group compared to the MASL group (*p* < 0.001). Additionally, ALT and AST enzyme levels were higher in the MASH group than in the MASL group (*p* < 0.003; *p* = 0.008, respectively). There were no significant differences between the groups in terms of lipid profile, glycemic parameters, intrahepatic triglycerides (IHTG), CRP levels, or liver size.

**Figure 1:**
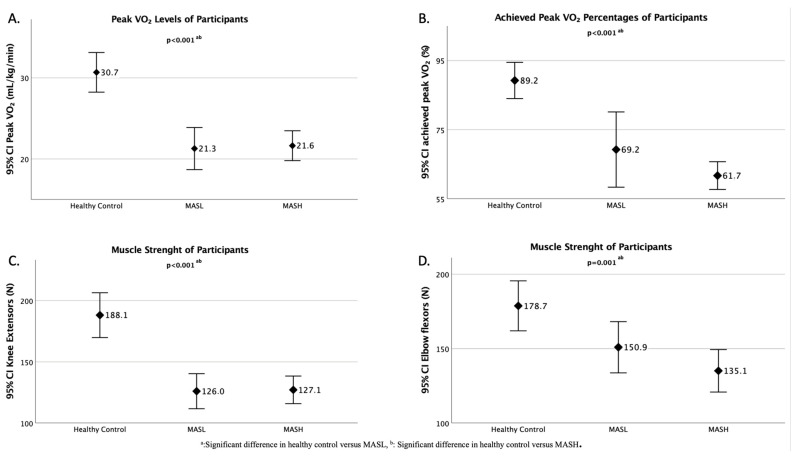
Peak VO_2_ levels of participation (**A**), Achieved peak VO_2_ percentages of participation (**B**), Knee extensors strength of participants (**C**), Elbow flexor strength of participants (**D**).

The VO_2_ levels were significantly lower in the MASL and MASH groups compared to the healthy control group, with the MASH group exhibiting the lowest levels among the groups (*p* < 0.001; Figure 1A). Figure 1B shows that the MASH and MASL groups achieved significantly lower percentages of the predicted peak VO_2_ compared to the healthy control group, with the MASH group achieving 62%, the MASL group achieving 69%, and the healthy individuals achieving 89% (*p* < 0.001). The knee extensor and elbow flexor strengths were significantly lower in the MASL and MASH groups compared to the healthy control group. However, there were no significant differences in these muscle groups between the MASL and MASH groups (*p* < 0.001 and *p* = 0.001, respectively; Figure 1C,D). While no significant differences were observed between the groups in terms of test termination (*p* = 0.192), participants in the MASH and MASL groups were more likely to stop due to fatigue and difficulty maintaining the required test pace compared to the healthy control group.

The participants’ muscle strength, physical performance, and patient-reported outcome measures are presented in Table 3. Appendicular muscle strength was significantly reduced in the MASL and MASH groups compared to the healthy controls, particularly in specific muscle groups like the shoulder flexors (*p* = 0.008) and elbow extensors (*p* = 0.049). The difference in elbow extensor strength approached statistical significance, which may be attributed to the sample size. Although there was no significant difference in total physical activity levels among the groups (*p* = 0.09), both MASLD and MASH patients engaged in less vigorous and moderate-intensity physical activities, with the MASH group completely abstaining from vigorous activities (*p* = 0.012 and *p* < 0.001, respectively). Regarding physical performance, TUG test results indicated that all participants scored below the 12 s threshold, suggesting no severe impairment. However, patients in the MASL and MASH groups had significantly longer TUG times compared to the healthy controls (*p* < 0.001). Fatigue levels were significantly higher in both the MASL and MASH groups compared to the healthy control group (*p* < 0.001), with no significant difference between the patient groups themselves. In terms of quality of life, the MASL and MASH groups reported lower scores in physical function, general health perception, and pain subdomains, with the MASH group exhibiting particularly poorer general health perception (*p* = 0.003). Regarding mental health, the HADS-Depression scores showed a significant difference between the healthy controls and the MASH group (*p* = 0.006), with the MASH group reporting higher levels of depression. No significant differences were found in HADS-Anxiety scores across the groups (*p* = 0.108). Overall, all participants’ mental health indicators remained within clinically acceptable ranges, indicating no severe anxiety or depression. After the maximal exercise test, systolic and diastolic blood pressure changes were within clinically acceptable limits for all participants, with no significant differences between the groups (*p* = 0.639 and *p* = 0.656, respectively).

Multiple linear regression analysis was conducted to examine the relationship between muscle strength and MASLD. All independent variables were included in the model, and variables that did not contribute were sequentially removed using a backward elimination approach. The model explained 60% of the variance in the dependent variable (R^2^ = 0.604). The results indicated that higher CRF levels were positively associated with muscle strength, while liver fat accumulation, elevated LDL levels, and depression emerged as potential predictors of sarcopenia. Although the association between AST levels and muscle strength was not statistically significant (*p* = 0.06), the results suggest a potential trend, with the 95% confidence interval ranging from −0.88 to 0.02; therefore, AST was removed from the model. Detailed findings are presented in Table 4.

## 4. Discussion

In our study, we investigated the relationship between muscle strength, aerobic capacity, physical performance, quality of life, emotional status, and disease severity in individuals diagnosed with MASLD. We found that as muscle strength increased in MASLD patients, liver fat accumulation, LDL levels, and depression decreased, while aerobic capacity improved. Additionally, reductions in muscle strength, CRF levels, and physical performance were observed in MASLD patients, particularly within the MASH population, where a lack of high-intensity physical activity, prolonged sitting times, increased fatigue, and depression levels were coupled with a diminished health perception. These findings parallel the functional and metabolic benefits provided by muscle strength [40]. This study provides the first data on muscle strength in individuals with MASLD, which may serve as a foundation for future research. Measurements taken with the manual muscle tester can be considered the initial reference values for this population; however, the validity and generalizability of these values should be confirmed in future studies involving larger and more diverse patient groups. Future research may expand on these findings to establish more reliable reference points.

The pathophysiological relationship between muscle tissue and MASLD is a complex process characterized by reciprocal interactions. In our study, we observed the effects of metabolic parameters, particularly LDL and IHTG, on muscle strength. Metabolic disorders such as insulin resistance and dyslipidemia can lead to lipotoxicity, accelerating the development of MASH and resulting in more severe liver complications [16]. Both IHTG and LDL are known to be closely associated with insulin resistance [41]. During exercise, myokines secreted by muscles exert anti-inflammatory effects, and muscle activity, by regulating energy metabolism and hormonal balance, enhances tissue sensitivity to insulin [42]. The inverse relationship between muscle strength and metabolic changes found in our study supports these results. Another finding was the increase in peak VO_2_ with enhanced muscle strength, highlighting the role of muscles in oxygen and energy metabolism. Reduced muscle strength limits oxygen consumption, leading to less efficient muscle function [43]. This suggests that in patients with metabolic diseases like MASLD, muscle strength not only deteriorates functional capacity but also impairs cardiorespiratory fitness.

Age-related changes in body composition can also affect the development of MASLD and MASH. As people age, an increase in fat mass and a loss of muscle mass are commonly observed [44]. These changes cannot be fully captured by general body metrics, such as BMI, as these measures only account for weight changes and overlook the distribution of fat and muscle tissue. This is particularly important in detecting sarcopenic obesity, which is often accompanied by metabolic factors such as insulin resistance, metabolic syndrome, diabetes, and hypertension [17]. Although our analysis revealed that BMI was significantly higher in the MASL and MASH groups compared to the control group, further regression analyses did not demonstrate a significant relationship between BMI and muscle strength in our study population. Notably, the ratio of increased visceral fat to decreased skeletal muscle mass is known to have a synergistic effect on insulin resistance and the risk of MASLD [45]. Therefore, future studies should consider muscle strength or visceral fat amount rather than BMI to better understand the impact of changes in muscle and fat mass on MASLD and MASH.

The relationship between chronic liver diseases and sarcopenia has become an increasingly prominent area of research. Clinical guidelines recommend assessing muscle strength to evaluate the risk of sarcopenia. However, despite the assignment of an ICD-10-CM code (M62.84) for sarcopenia, a widely accepted diagnostic criterion is still lacking [44]. Several studies in the literature have examined the relationship between MASLD and sarcopenia in terms of muscle strength [28,46,47,48]. These studies generally report a negative relationship between muscle strength and MASLD. In our study, we referenced the quadriceps femoris muscle, which plays a crucial role in daily activities (such as walking and stair climbing) and is considered a significant predictor for diagnosing and monitoring sarcopenia [24,49]. Our findings indicate a 29% reduction in knee extension strength in the MASL group and a 35% reduction in the MASH group. Although no statistically significant differences were observed in other muscle groups, individuals in the MASL and MASH groups exhibited lower muscle strength compared to healthy controls. Our results for the control group are consistent with a previous study that investigated isometric muscles [50]. Additionally, although our participants did not have T2DM or obesity, the decrease in muscle strength could be an early clinical manifestation of insulin resistance, one of the key mechanisms underlying MASLD.

One of the parameters examined in our study was the relationship between aerobic capacity and MASLD. It is well established that changes in mitochondrial function and oxidative stress negatively impact CRF by impairing oxygen utilization and cellular respiration [51]. The increased oxidative stress, a result of mitochondrial dysfunction, impairs fatty acid oxidation, further exacerbating mitochondrial impairment. This, in turn, reduces the efficiency of oxygen utilization in skeletal muscles, compromising aerobic capacity. Furthermore, oxidative stress decreases mitochondrial quantity, which negatively affects ATP synthesis and oxidative phosphorylation-key processes for energy production during physical exertion [52,53]. As a result, the overall aerobic fitness and cardiovascular health of individuals with MASLD are significantly compromised. Furthermore, research indicates a correlation between MASLD and CRF, demonstrating an elevated risk of cardiovascular disease in patients with MASLD, as well as reports of increased fatigue levels. Our findings corroborate existing research in this area [7,54,55]. Unlike previous studies, we assessed CRF using the ISWT. The 6 min walk test is frequently preferred for its simplicity; however, the progressively increasing pace of the ISWT produces physiological responses that more closely parallel those obtained from CPET [31]. The ISWT is also a simple assessment that can function as a prognostic marker in metabolic-related diseases [56]. Consistent with the literature, which highlights a mild but significant decline in physical performance among individuals with metabolic dysfunction [57], our findings indicate that impairments in physical performance are commonly associated with advanced sarcopenia.

Our study revealed a negative relationship between muscle strength and depression. This finding aligns with a meta-analysis of 87,508 adults that investigated the association between muscle strength and depression, revealing a significant correlation (95% CI: 0.80 to 0.89) [58]. This finding highlights the potential importance of incorporating approaches that support emotional well-being in interventions aimed at enhancing muscle strength. Additionally, we observed a negative relationship between LDL levels and muscle strength. Although Lee Jun-Hyuj et al. [59] demonstrated a similar relationship between LDL levels and muscle mass, our study’s sample size is too small to generalize these findings to the broader population. Further research in this area is warranted to clarify these associations.

In our study, we were unable to identify a relationship between liver fibrosis and muscle strength. However, recent studies have shown that chronic inflammation and insulin resistance, which are commonly observed alongside liver fibrosis, are significant contributors to muscle loss [51]. Additionally, elevated triglyceride levels are believed to be associated with sarcopenia [60]. The inability to demonstrate these relationships in our study may be due to insufficient statistical power. Although we included challenging physical assessments, such as maximal aerobic capacity evaluation and maximal muscle strength measurements, we did not encounter any adverse events. Finally, in our model examining the relationship between muscle strength and MASLD biomarkers, AST was excluded due to borderline significance (*p* = 0.06). Considering that the AST:ALT ratio may indicate progression to advanced stages of MASLD, particularly to MASH [61], future studies in patients with advanced fibrosis should validate these associations.

Given that the progression of MASLD can lead to severe liver complications requiring surgical intervention, optimizing patients’ physical and metabolic health prior to surgery is crucial. Prehabilitation programs, which have been shown to enhance muscle strength, aerobic capacity, and overall physical performance, can reduce postoperative complications and improve recovery [62,63,64]. Our findings, demonstrating the positive impact of muscle strength on metabolic parameters and liver health in MASLD patients, align with these principles. Specifically, the observed improvements in aerobic capacity and reductions in liver fat accumulation and LDL levels with increased muscle strength suggest that prehabilitation programs could benefit MASLD patients. Implementing such programs in clinical practice may not only improve liver health but also reduce hospital stay duration and enable patients to regain functional independence more quickly. Future studies should explore the efficacy of tailored prehabilitation interventions in MASLD populations, with a particular focus on their impact on disease progression and patient outcomes.

### Strengths and Limitations of the Study

This study offers valuable insights into the relationship between muscle strength, metabolic parameters, and liver disease in individuals with MASLD. A notable strength of the study is its comprehensive approach, encompassing various factors such as muscle strength, aerobic capacity, physical performance, and emotional status. Additionally, the study provides the first data on muscle strength in individuals with MASLD, contributing significantly to the existing body of literature. The use of a manual muscle tester for assessing muscle strength is also a strength as it establishes initial reference values that could be beneficial for future clinical studies and practices.

However, the study is not without limitations. The relatively small sample size limits the generalizability of the findings, especially when examining relationships between muscle strength and more complex factors such as liver fibrosis. This limitation may also explain the inability to detect some significant relationships, such as between muscle strength and liver fibrosis. Additionally, the cross-sectional design of the study restricts the ability to draw conclusions about causality or long-term changes in muscle strength and MASLD progression. Moreover, the participants in our study did not have T2DM or obesity, which means the sample represents only a small subset of the broader MASLD population. As such, the findings may not be fully applicable to individuals with more advanced stages of MASLD or those with comorbid conditions. Therefore, future research should focus on investigating the role of muscle strength and insulin resistance across the entire MASLD spectrum, including its more advanced stages and in diverse patient populations. Given the absence of established cut-off values for appendicular muscle strength to determine sarcopenia prevalence, future studies could explore alternative markers or develop standardized criteria to better assess sarcopenia in clinical populations.

## 5. Conclusions

This study suggests that muscle strength is associated with key metabolic parameters, including LDL levels, IHTG content, and aerobic capacity, which may be important for the development of MASLD and its progression to more severe liver conditions, such as MASH. These findings indicate that reducing or at least delaying the onset of muscle loss could be necessary for the prevention of metabolic disorders involving the liver. Therefore, interventions aimed at maintaining or enhancing muscle strength in individuals with MASLD may play a crucial role in mitigating liver damage and improving overall metabolic health. Further research with larger sample sizes and longitudinal designs is required to confirm these findings and explore the potential therapeutic implications of preserving muscle function in MASLD patients.

## Figures and Tables

**Table 1:** Demographic and clinical characteristics of participants.

Characteristics	Healthy Control	MASL	MASH	Between Group
	(n = 28)	(n = 15)	(n = 17)	*p*-Value
Age (years), mean ± SD	43.9 ± 10.2	45.5 ± 13.3	46.1 ± 6.3	0.758
Gender, n (%)				0.197
Male	20 (71)	8 (53)	14 (82)	
Female	8 (29)	7 (47)	3 (18)	
BMI (kg/m^2^), mean ± SD	25.7 ± 3	28.5 ± 3.3	29.4 ± 2.6	<0.001
Smoking status, n (%)				0.572
Never smoker	18 (64)	9 (60)	14 (82)	
Previous smoker	1 (4)	1 (7)	1 (6)	
Current smoker	9 (32)	5 (33)	2 (12)	
Comorbidities ^, n (%)				0.550
Arterial hypertension	5 (18)	4 (27)	7 (41)	
Thyroid diseases	3 (11)	1 (7)	0 (0)	
Chronic respiratory diseases	2 (7)	0 (0)	2 (12)	
Other	3 (11)	0 (0)	1 (6)	

Note: ^ Multiple diseases exist and the percentages are approximated.

**Table 2:** The clinical characteristics of patients with MASLD.

Clinical Aspects	MASL (n = 15)	MASH (n = 17)	*p*-Value
Disease duration (years)	5.4 ± 4	4 ± 3.5	0.280
MRI-PDFF IHTG (%)	14.6 ± 6.9	16.4 ± 7.8	0.495
Liver stiffness value (kPa)	2.2 (0.2)	2.7 (0.5)	0.001
Liver size (cm)	16.2 (1.7)	17.4 (2.5)	0.189
CRP (mg/dL) (0–0.8)	0.4 (0.5)	0.4 (0.3)	0.794
ALT (U/L) (<50)	41.2 ± 12.7	65.6 ± 26.4	0.003
AST (U/L) (<50)	27.7 ± 5.4	37.4 ± 12.6	0.008
GGT (U/L) (<55)	42 (41)	53 (40.5)	0.193
HDL (mg/dL) (>40)	53 (10)	45 (15)	0.056
LDL mg/dL (<130)	143 ± 25	133.9 ± 34.3	0.402
Triglyceride (mg/dL) (<200)	153 ± 64.5	163.8 ± 71.8	0.658
Fasting Glucose (mg/dL) (100–125)	102 (25)	98 (20)	0.478
Fasting Insulin (uIU/mL) (1.9–23)	14.1 ± 7	15 ± 8	0.736
HbA1c (%) (3.5–5.6)	5.6 (0.5)	5.8 (0.5)	0.278

MRI-PDFF: magnetic resonance imaging-proton density fat fraction; IHTG: intrahepatic triglyceride; CRP: C-reactive protein; ALT: alanine aminotransferase; AST: aspartate aminotransferase; GGT: gamma-glutamyl transferase; HDL: high-density lipoprotein; LDL: low-density lipoprotein; HbA1c: glycated hemoglobin.

**Table 3:** Outcome measurements of participants with MASLD and healthy controls.

Parameters	Healthy Control	MASL	MASH	Between Group
	(n = 28)	(n = 15)	(n = 17)	*p*-Value
Predicted peak VO_2_ (mL/kg/min)	36.7 (10.1)	32 (20.2)	36.9 (5.6)	0.675
ISWT Distance (m)	722.4 ± 170.9	526.6 ± 92.4	509.7 ± 61.8	<0.001 ^ab^
Muscle Strength (Newton)				
Shoulder flexors	140.3 ± 30.1	122.3 ± 22.5	117 ± 16.3	0.008 ^b^
Shoulder extensors	106.8 ± 23.1	106 ± 15.3	106.9 ± 19.5	0.758
Elbow extensors	152.6 ± 39.3	127.9 ± 25.9	133.1 ± 31.4	0.049
Hip flexors	155.2 ± 41.3	130.5 ± 20.2	135.8 ± 28.8	0.124
Hip extensors	136.5 (55.3)	116 (32)	125.3 (40.8)	0.51
Knee flexors	134.5 (58.9)	98.3 (16)	113.2 (25.8)	0.276
IPAQ				
Vigorous PA (MET-min/week)	0 (90)	0 (0)	0 (0)	0.012 ^b^
Moderate PA (MET-min/week)	180 (720)	0 (0)	0 (0)	<0.001 ^ab^
Walking (MET-min/week)	1386 (1934.6)	1386 (1617)	924 (1732.5)	0.545
Total PA (MET-min/week)	2046 (1846.5)	1386 (1617)	1113 (1732.5)	0.090
Sitting (min/week)	368.6 ± 128.3	376 ± 165.8	487.1 ± 137.3	0.021 ^b^
Timed Up and Go (s)	4.9 ± 0.7	6.7 ± 1	6.8 ± 1.1	<0.001 ^ab^
Fatigue Severity Scale	2.5 (1.3)	3.4 (2.9)	4.1 (2.7)	<0.001 ^ab^
Short Form-36				
Physical Functioning	100 (5.6)	88.9 (27.8)	88.9 (25)	0.003 ^ab^
Role-Physical	100 (25)	100 (100)	50 (100)	0.078
Bodily Pain	74.1 ± 15	58 ± 17.6	57.6 ± 15.8	0.001 ^ab^
General Health	77.5 ± 19	69 ± 20.1	60 ± 24.4	0.030 ^b^
Vitality	100 (12.5)	87.5 (25)	100 (37.5)	0.189
Social Functioning	66.7 (33.3)	66.7 (100)	66.7 (100)	0.364
Role-Emotional	90 (17.5)	100 (10)	100 (45)	0.372
Mental health	76.6 ± 13.7	66.4 ± 17.9	62.4 ± 24.2	0.033
Hospital Anxiety and Depression Scale				
HADS-Anxiety	3 (2)	5 (7)	5 (5)	0.108
HADS-Depression	2 (3)	4 (7)	4 (4)	0.006 ^b^
Change in Blood Pressure				
ΔSBP (mmHg)	25.9 ± 11.9	22.1 ± 14.8	25.1 ± 12.5	0.639
ΔDBP (mmHg)	4.5 ± 8.1	2.5 ± 7.4	5.1± 9.7	0.656

Note: ^a^: Significant difference in healthy control versus MASL, ^b^: significant difference in healthy control versus MASH.

**Table 4:** Association between muscle strength and clinical parameters in MASLD patients.

Variable	Odds Ratio (OR)	95% Confidence Interval (CI)	*p*-Value
Peak VO_2_	0.531	1.27 to 3.47	<0.001
HADS-Depression	−0.384	−3.10 to −0.74	0.003
IHTC	−0.287	−1.31 to −0.11	0.023
LDL	−0.286	−0.02 to −0.33	0.03

## Data Availability

The data presented in this study are available on request from the corresponding author.

## Supplementary Materials

The following supporting information can be downloaded at: https://www.mdpi.com/article/10.3390/medicina61020247/s1, Table S1: Comparison of NAFLD and MASLD diagnostic criteria; Table S2: Cardiometabolic risk factors of the patients included in the study.

## Abbreviations

MASLD: metabolic dysfunction-associated steatotic liver disease; NAFLD: non-alcoholic fatty liver disease; MASL: metabolic dysfunction-associated steatotic liver; MASH: metabolic dysfunction-associated steatohepatitis; IL: interleukin; TNF: Tumor necrosis factor; CRF: cardiorespiratory fitness; MRI-PDFF: magnetic resonance imaging-proton density fat fraction; MRE: magnetic resonance elastography; BMI: body mass index; T2DM: type 2 diabetes mellitus; HbA1c: Glycated hemoglobin; ALT: alanine aminotransferase; AST: aspartate aminotransferase; GGT: gamma-glutamyl transferase; HDL: high-density lipoprotein; LDL: low-density lipoprotein; CRP: C-reactive protein; ISWT: incremental shuttle walk test; CPET: cardiopulmonary exercise testing; TUG: timed up and go; IPAQ: the international physical activity questionnaire; FSS: fatigue severity scale; SF-36: short form-36; HADS: hospital anxiety and depression scale; IHTG: intrahepatic triglyceride; Peak VO_2_: the highest rate of oxygen uptake; PA: physical activity; MET: metabolic equivalent of task; SBP: systolic blood pressure; DBP: diastolic blood pressure.
