# Supplementary material for: Muscle Strength and Cardiovascular Health in MASLD: A Prospective Study
Source: Medicina (Kaunas). 2025 Feb 1;61(2):247. doi: 10.3390/medicina61020247 (PMC11857117; doi:10.3390/medicina61020247)
Supplement: Supplementary file 1 [file medicina-61-00247-s001.zip › medicina-3369581-supplementary.pdf]

Muscle Strength and Cardiovascular Health in MASLD: A Prospective Study

Birgül Fatma Kumbaroğlu[1]*, Yasemin Hatice Balaban[2], Tülin Düger[1]

[1] Faculty of Physical Therapy and Rehabilitation, Hacettepe University, Ankara, Turkey.

[2] Faculty of Medicine, Department of Gastroenterology, Hacettepe University, Ankara, Turkey.

Table of Contents

Comparison of NAFLD- MASLD diagnostic criteria

Cardiometabolic risk factors of the patients included in the study

Table S1. The comparison of NAFLD and MASLD diagnostic criteria (1-3)

Parameter	NAFLD Diagnostic Criteria	MASLD Diagnostic Criteria (2023)
**Updated Terminology**	NAFLD (Non-Alcoholic Fatty Liver Diseases) and NASH (Non-Alcoholic Steatohepatitis)	MASLD (Metabolic Dysfunction-Associated Steatotic Liver Disease) and MASH (Metabolic-Associated Steatohepatitis)
**Hepatic Steatosis**	>5% hepatic fat accumulation, confirmed by imaging OR biopsy	>5% hepatic fat accumulation, confirmed by imaging AND biopsy
**Dual Etiology**	Alcohol related or other liver diseases must be excluded	Associated alcohol related or other liver diseases may be present

**Metabolic Risk Factors**	Not required; however, obesity, diabetes, and metabolic factors are frequently observed	Required: At least one cardiometabolic risk factor BMI: ≥ 25 kg/m² or Waist Circumference: > 94 cm for men, > 80 cm for women<br>Fasting Blood Glucose: ≥ 100 mg/dL, or Post-load Glucose (after two-hour glucose tolerance test): ≥ 140 mg/dL, or HbA1c: ≥ 5.7% (39 mmol/mol), or a diagnosis of Type 2 diabetes, or current diabetes treatment<br>Blood Pressure: ≥ 130/85 mmHg or current antihypertensive treatment<br>Plasma Triglycerides: ≥ 150 mg/dL or under lipid-lowering treatment<br>Plasma HDL-Cholesterol: ≤ 40 mg/dL for men and ≤ 50 mg/dL for women, or under lipid-lowering treatment
**Alcohol Intake**	<20 g/day for females, <30 g/day for males	Same limits apply, but with a distinct category (MetALD) for higher alcohol intake, beyond MASLD criteria

Table S2. Cardiometabolic risk factors in patients diagnosed with NAFLD included in the study

**Patient id**	**BMI**	**Blood Glucose**	**Blood Pressure**	**Triglyceride**	**HDL**
1.	✓	✓	✓	X	X
2.	✓	X	✓	X	X
3.	✓	✓	X	✓	X
4.	✓	✓	✓	X	X
5.	✓	✓	X	X	X
6.	✓	X	X	X	X
7.	✓	✓	✓	✓	X
8.	✓	X	X	✓	X
9.	✓	✓	X	X	✓
10.	✓	✓	✓	X	X
11.	✓	✓	✓	X	X
12.	✓	✓	X	X	✓
13.	✓	✓	✓	✓	✓
14.	✓	✓	X	✓	X
15.	✓	✓	X	✓	✓
16.	✓	X	X	✓	X
17.	✓	✓	X	X	X
18.	✓	X	✓	X	✓

19.	✓	X	X	✓	X
20.	✓	X	X	X	X
21.	X	✓	X	X	X
22.	✓	✓	X	X	X
23.	✓	✓	✓	✓	X
24.	✓	✓	X	X	✓
25.	✓	✓	X	✓	X
26.	✓	✓	X	X	X
27.	✓	✓	✓	✓	X
28.	✓	✓	X	✓	X
29.	✓	X	✓	X	X
30.	✓	✓	X	✓	X
31.	X	X	X	✓	X
32.	✓	✓	X	✓	X

Note: A checkmark indicates the presence of a cardiometabolic factor, while an X indicates its absence.

References

1. Chen L, Tao X, Zeng M, Mi Y, Xu L. Clinical and histological features under different nomenclatures of fatty liver disease: NAFLD, MAFLD, MASLD and MetALD. Journal of Hepatology. 2024;80(2):e64-e6.

2. Ramírez-Mejía MM, Méndez-Sánchez N. What Is in a Name: from NAFLD to MAFLD and MASLD—Unraveling the Complexities and Implications. Current Hepatology Reports. 2023;22(4):221-7.

3. Loomba R, Wong VWS. Implications of the new nomenclature of steatotic liver disease and definition of metabolic dysfunction-associated steatotic liver disease. Alimentary pharmacology & therapeutics. 2024;59(2):150-6.
